# The Effect of Xevinapant Combined with Ionizing Radiation on HNSCC and Normal Tissue Cells and the Impact of Xevinapant on Its Targeted Proteins cIAP1 and XIAP

**DOI:** 10.3390/cells12121653

**Published:** 2023-06-17

**Authors:** Julia Fleischmann, Laura S. Hildebrand, Lukas Kuhlmann, Rainer Fietkau, Luitpold V. Distel

**Affiliations:** 1 Department of Radiation Oncology, Universitätsklinikum Erlangen, Friedrich-Alexander-Universität Erlangen-Nürnberg, 91054 Erlangen, Germany; fl.julia@t-online.de (J.F.); laura.hildebrand@uk-erlangen.de (L.S.H.); lukas.kuhlmann@uk-erlangen.de (L.K.); rainer.fietkau@uk-erlangen.de (R.F.); 2 Comprehensive Cancer Center Erlangen-Europäische Metropolregion Nürnberg (CCC ER-EMN), 91054 Erlangen, Germany

**Keywords:** ionizing radiation, interactions, Xevinapant, apoptosis, inhibitor, cIAP1/2, XIAP, head and neck squamous cell carcinoma, targeted therapy

## Abstract

The poor prognosis of HNSCC is partly due to treatment resistance. The SMAC mimetic Xevinapant is a promising new approach to targeted cancer therapy. Xevinapant inhibits cIAP1/2 and XIAP, leading to apoptosis, necroptosis and inhibition of prosurvival signaling. Combining Xevinapant with IR could improve therapeutic potential. The effect of Xevinapant in combination with IR on HNSCC and healthy tissue cells was investigated. Cell growth, cell death, clonogenic survival and DNA double-strand breaks (DSBs) were studied, and intracellular cIAP1 and XIAP levels were evaluated. Xevinapant had cytostatic and cytotoxic, as well as radiosensitizing, effects on the malignant cells, while healthy tissue cells were less affected. Apoptotic and necrotic cell death was particularly affected, but the increase in residual DSBs and the reduced survival implied an additional effect of Xevinapant on DNA damage repair and other cell inactivation mechanisms. cIAP1 and XIAP levels varied for each cell line and were affected by Xevinapant and IR treatment. There was an association between higher IAP levels and increased cell death. Xevinapant appears to be a potent new drug for HNSCC therapy, especially in combination with IR. IAP levels could be an indicator for impaired DNA damage repair and increased susceptibility to cellular stress.

## 1. Introduction

Head and neck squamous cell carcinoma (HNSCC) is a primary malignant tumor that arises from epithelial cells mainly in the oral cavity, the pharynx and the larynx, and to a lesser extent, in the nasal cavity or paranasal sinuses [1]. It is the 7th most common cancer worldwide, with an estimated incidence of over 870,000 new cases diagnosed in 2020, and it is continuing to rise [2]. Two major risk factors for the development of HNSCC are tobacco and alcohol use. Tobacco and alcohol are mainly responsible for tumors in the oral cavity and the larynx, accounting for approximately 70–80% of HNSCC cases [3,4]. Another major risk factor is an infection with human papillomavirus (HPV). Over the past few decades, the prevalence of HPV-associated HNSCC has increased significantly. These cases are classified as HPV-positive tumors and occur mainly in the oropharynx (>70%), mostly due to infection with the high-risk HPV-16 type. Patients with HPV-positive HNSCC are on average younger and have a better clinical prognosis than patients with HPV-negative HNSCC [5,6,7,8,9,10].

Therapy of both HPV-positive and HPV-negative HNSCC involves surgery, radiotherapy (RT) or chemoradiotherapy (CRT) [11,12,13]. A few targeted therapies have been approved, such as the checkpoint inhibitors pembrolizumab and nivolumab for recurrent or metastatic HNSCC, or the epidermal growth factor receptor (EGFR) inhibitor cetuximab for locally advanced HNSCC [14,15,16,17]. Despite treatment, the prognosis for HNSCC remains poor, and more than half of the patients with locally advanced HNSCC will develop locoregional recurrence or distant metastases [18,19]. One of the reasons for poor long-term survival in patients with HNSCC is the resistance of cancer cells to treatment, partly due to genetic heterogeneity and different genetic alterations that affect multiple intracellular signaling pathways [20,21]. The apoptotic pathway is often deregulated in several tumors, including HNSCC, with overexpression of inhibitors of apoptosis proteins (IAPs), leading to evasion of apoptosis [22,23,24,25,26]. IAPs, including cellular IAP 1 and 2 (cIAP1/2) or X-linked IAP (XIAP), are a family of antiapoptotic proteins, which regulate cell death and apoptosis. XIAP is a direct inhibitor of caspases in both the intrinsic and the extrinsic apoptotic pathways [27,28,29]. cIAP1/2 inhibits the formation of proapoptotic complexes in the extrinsic apoptotic pathway, leading to inhibition of apoptosis and necroptosis, another form of programmed cell death [30,31,32,33,34]. Further, cIAP1/2 plays a role in the NF-κB signaling pathway, which regulates apoptosis und immune response [35,36,37,38]. An endogenous inhibitor of IAPs is the second mitochondria-derived activator of caspase (SMAC), also known as DIABLO. SMAC is a mitochondrial protein that is released into the cytosol in response to apoptotic stimuli. SMAC binds IAPs and antagonizes their inhibitory activity, resulting in an activation of caspases and apoptotic cell death [39,40,41].

Over the past two decades, SMAC mimetics have emerged as a novel approach to targeted cancer therapy. These small molecules mimic the effect of SMAC in antagonizing IAPs and facilitating apoptotic and necroptotic cell death. They directly inhibit XIAP, while their inhibitory effect on cIAP1/2 is mediated by autoubiquitination and proteasomal degradation of cIAP1/2 [36,37,42,43,44,45]. No SMAC mimetics are yet approved as cancer therapeutics, but several are currently undergoing clinical trials, including Xevinapant (also known as Debio 1143, AT-406, SM-406) [46]. Xevinapant is an orally available monovalent SMAC mimetic, which targets cIAP1, cIAP2 and XIAP, and therefore enhances apoptotic or necroptotic cell death. In addition, the inhibition of cIAP1/2 by Xevinapant amplifies antitumor immune responses via NF-κB signaling [47,48,49]. Xevinapant has shown good antitumor effects in several preclinical studies [47,50,51,52,53,54,55,56] and is currently in one phase 1, one phase 2 and two phase 3 clinical trials [57]. The TrilynX study is evaluating Xevinapant + CRT versus Placebo + CRT in patients with locally advanced HNSCC, measuring event-free survival as the primary endpoint of the study [58]. The X-ray vision study is evaluating Xevinapant + RT versus Placebo + RT in patients with resected HNSCC, who are ineligible for Cisplatin, with disease-free survival as the primary outcome measure [59]. 

The effects of Xevinapant are not yet fully understood, and most of the preclinical data focus on Xevinapant in combination with chemotherapy, while there are very few data on treatment with Xevinapant in combination with RT, particularly in HNSCC in vitro. Therefore, this study investigated the therapeutic potential and effect of Xevinapant alone and in combination with ionizing radiation (IR) on various HNSCC cell lines, as well as on healthy tissue cells. In addition, the expression of the Xevinapant target proteins cIAP1 and XIAP was evaluated as a potential marker of sensitivity or resistance to Xevinapant treatment. 

## 2. Materials and Methods

### 2.1. Cell Culture and Inhibitor

Nine cell lines were used in total: seven HNSCC cell lines (Cal33, CLS-354, Detroit 562, HSC4 and RPMI-2650 as HPV-negative and UD-SSC-2 and UM-SSC-47 as HPV-positive HNSCC cell lines), one cell line of healthy oral mucosa (01-GI-SBL) and human skin fibroblasts (SBLF9). Cal33, HSC4, UD-SSC-2 and UM-SSC-47 were provided by Dr. Thorsten Rieckmann (University Medical Center, Hamburg-Eppendorf, Germany), CLS-354, Detroit 562, and RPMI-2650 was purchased from CLS cell lines service GmbH (Eppelheim, Germany). 01-GI-SBL and SBLF9 were obtained from two different healthy donors. The ethical approval was obtained from the ethics committee of the medical faculty of the Friedrich-Alexander-Universität Erlangen-Nürnberg (204_17 BC). The HNSCC cell lines and the oral mucosa cell line were cultured in Dulbecco’s modified Eagle´s Medium (DMEM; PAN-Biotech, Aidenbach, Germany), supplemented with 10% fetal bovine serum (FBS; Sigma Aldrich, St. Louis, MI, USA) and 1% penicillin/streptomycin (Thermo Fisher scientific, Waltham, MA, USA). SBLF9 was cultivated in F-12 medium (Thermo Fisher scientific, Waltham, MA, USA) with 15% FBS, 2% nonessential amino acids (NEA; Bio&SELL GmbH, Feucht, Germany) and 1% penicillin/streptomycin. All cells were incubated at 37 °C in a humidified 5% CO_2_ atmosphere. SMAC mimetic Xevinapant (Selleck Chemicals LLC, Houston, TX, USA) was dissolved in dimethyl sulfoxide (DMSO; Carl Roth GmbH + Co. KG, Karlsruhe, Germany) and stored at −80 °C. The required aliquots were freshly thawed before each experiment. 

### 2.2. Live-Cell Microscopy 

Cells were seeded into a 24-well-plate with a density of 30,000 to 40,000 cells and 0.8 mL medium per well. Immediately afterwards, monitoring began with a 24-channel microscope in the incubator, and 1 image per hour was acquired (zenCell owl, innoME GmbH, Espelkamp, Germany). Cells were left to settle for 24 h before Xevinapant was added in different concentrations. Then, 3 h after the application of Xevinapant, cells were irradiated with a dose of 2 Gy. After 48 h of treatment with Xevinapant, the medium was replaced with drug-free medium. Monitoring continued until the surface of the wells was fully overgrown. The number of cells over time was captured by ZenCell Owl software, and growth curves were generated. To evaluate the doubling time, an exponential growth equation was fitted to the data:

y=y0×exp(k×x)

where y0 is the cell number at the start of monitoring (x = 0), k is the rate constant and x is the time in hours. Doubling time is computed as $\ln\left(2\right)/k$.

### 2.3. Colony Formation Assay

An adequate number of cells were seeded into 60 mm petri dishes containing 3 mL fresh medium and incubated for 24 h. Then, the cells were treated with Xevinapant, and half of them were additionally irradiated with a 2 Gy dose 3 h later. Medium was exchanged after a further incubation period of 48 h. Cells were incubated for 10 to 14 days, until colonies were formed. Afterwards, cells were stained with methylene blue (Carl Roth GmbH + Co. KG, Karlsruhe, Germany) for 30 min at room temperature, and colonies containing more than 50 cells were counted semiautomatically with an image analyzing software (Biomas, Erlangen, Germany). Plating efficiency (PE) and survival fraction (SF) were calculated. 

### 2.4. Cell Death Analysis by Flow Cytometry

Cells were seeded into T25 flasks, with a density of 100,000 cells per flask. After settling for 48 h, the medium was exchanged by a serum-reduced medium containing only 2% FBS. Next, cells were treated with Xevinapant, and 3 h later, half of them were irradiated with 2 Gy of IR. Subsequently, 48 h after the treatment with Xevinapant, cells including the supernatant were harvested and stained using 10 µL equal parts AnnexinV APC and 7AAD (BD, Franklin Lakes, NJ, USA), then cells were incubated on ice for 30 min. The staining agents were removed before the cells were centrifuged and resuspended in cold Ringer´s solution (Fresenius Kabi AG, Bad Homburg, Germany). Flow cytometry analysis was performed with the Cytoflex flow cytometer (Cytoflex, Beckman Coulter, Brea, CA, USA), and the data were analyzed using the Kaluza analysis software (Beckman Coulter, Brea, CA, USA).

### 2.5. Immunostaining Assay

Cells were seeded into 8-well rubber chambers on microscope slides at a density of 30,000 cells and 0.8 mL medium per chamber and were incubated for 48 h. Medium was then exchanged, Xevinapant was added to the cells, and 3 h later, an IR dose of 2 Gy was applied. Another 48 h after application of the drug, cells were fixed with 4% formaldehyde solution (Sigma Aldrich, St. Louis, MI, USA) and blocked with 1% bovine serum albumin (BSA, SERVA Electrophoresis GmbH, Heidelberg, Germany) overnight at 4 °C. Cells were then stained with either cIAP1 (Invitrogen, Thermo Fisher scientific, Waltham, MA, USA) 1:50, XIAP (Invitrogen, Thermo Fisher scientific, Waltham, MA, USA) 1:100 and Ki-67 (Invitrogen, Thermo Fisher scientific, Waltham, MA, USA) 1:50 or with γH2AX (BioLegend, San Diego, CA, USA) 1:2500 and Ki-67 1:100. All primary antibodies were diluted in 1% BSA and stained overnight at 4 °C. Subsequently, secondary antibodies Alexa fluor 488 chicken antigoat (Thermo Fisher scientific, Waltham, MA, USA), Alexa fluor 555 donkey antirabbit (Thermo Fisher scientific, Waltham, MA, USA), Alexa fluor 555 donkey antimouse (molecular probes, Thermo Fisher scientific, Waltham, MA, USA) and Alexa fluor 647 donkey antirat (Abcam, Cambridge, UK), diluted 1:200 in 1% BSA, were applied and incubated for 90 min at room temperature. Cells were stained with DAPI and covered with Vectashield (Vector laboratories, Newark, CA, USA). Images were acquired with a fluorescence microscope (Axioplan 2; Zeiss, Göttingen, Germany) and slide scanning software Metafer 4 (metasystems, Altlussheim, Germany). 

### 2.6. Statistical Analysis

Graphs were generated using Microsoft Excel 2016 (Microsoft Corporation, Redmond, WA, USA) and Graphpad Prism 9 (Graphpad software, San Diego, CA, USA). A one-tailed Mann–Whitney U test was used to determine significance with a significance level of *p* ≤ 0.050.

## 3. Results

**Figure 1:**
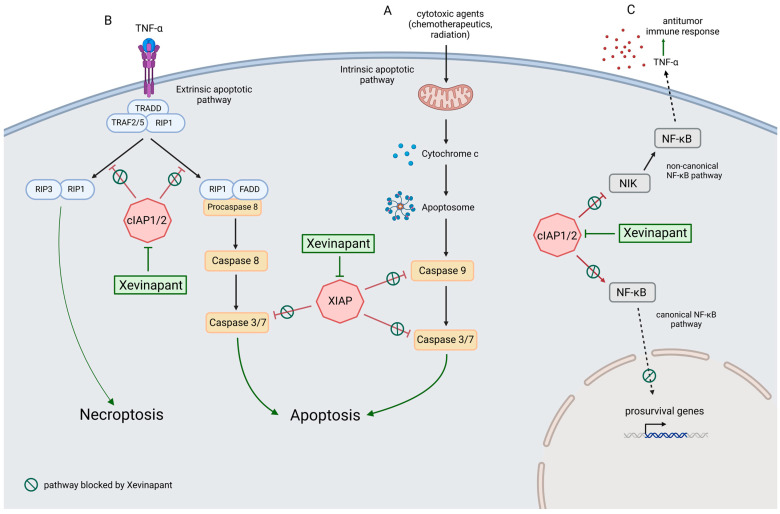
Xevinapant mode of action through blocking cIAP1/2 and XIAP: (**A**) In the intrinsic apoptotic pathway, cytochrome c is released from the mitochondria into the cytosol in response to apoptotic stimuli that cause intracellular stress or DNA damage. Cytochrome c molecules associate with Apaf-1 (apoptotic protease activating factor 1) to form the apoptosome. This leads to activation of caspase 9, followed by activation of caspases 3 and 7, resulting in apoptosis. XIAP inhibits apoptosis by blocking caspases 9, 3 and 7. Xevinapant inhibits XIAP, allowing the release of caspase activity and promotion of apoptotic signaling. (**B**) In the extrinsic pathway, death receptor ligands such as TNF-α stimulate apoptosis by binding to their membrane receptors. This leads to the assembly of TRADD (TNF receptor type 1-associated death domain protein), TRAF2/5 (TNF receptor associated factor) and protein kinase RIP1. In the presence of cIAP1/2, RIP1 is ubiquitylated, and activation of the apoptotic cascade is inhibited. When cIAP1/2 is blocked by Xevinapant, RIP1 is deubiquitylated and forms a complex with FADD (Fas-associated protein with death domain) and procaspase 8. This leads to activation of caspase 8, followed by caspases 3 and 7, and apoptosis takes place. Alternatively, in the absence of caspase 8, deubiquitylated RIP1 can form a complex with RIP3, called the necrosome. This promotes necrotic signaling and results in necroptosis, a programmed necrotic cell death. (**C**) cIAP1/2 blocks the noncanonical NF-κB pathway and enables canonical NF-κB signaling. Xevinapant inhibits cIAP1/2 downstream of the TNF-receptor, leading to activation of NIK and noncanonical NF-κB signaling. Proinflammatory cytokines such as TNF-α are released and the antitumor immune response of the tumor microenvironment is stimulated. In addition, Xevinapant inhibits the transcription of prosurvival genes by blocking the canonical NF-κB pathway (modified after [30,31,32,33,34,35,36,37,38,39,40,41,43,44,45,60,61,62] and created with BioRender.com, accessed on 28 April 2023).

SMAC mimetic Xevinapant inhibits the inhibitor of apoptosis proteins cIAP1/2 and XIAP and affects three different cell signaling pathways (Figure 1). In this study, the effect of Xevinapant alone and in combination with IR on nine different cell lines was investigated. Seven cell lines were HNSCC cell lines (Cal33, CLS-354, Detroit 562, HSC4, RPMI-2650, UD-SSC-2, UM-SSC-47), one was a healthy oral mucosa cell line (01-GI-SBL), and one was a skin fibroblast cell line (SBLF9). In addition, the expression levels of cIAP1 and XIAP, two targets of Xevinapant, were analyzed.

### 3.1. Determination of a Relevant Xevinapant Concentration and Growth Inhibition

**Figure 2:**
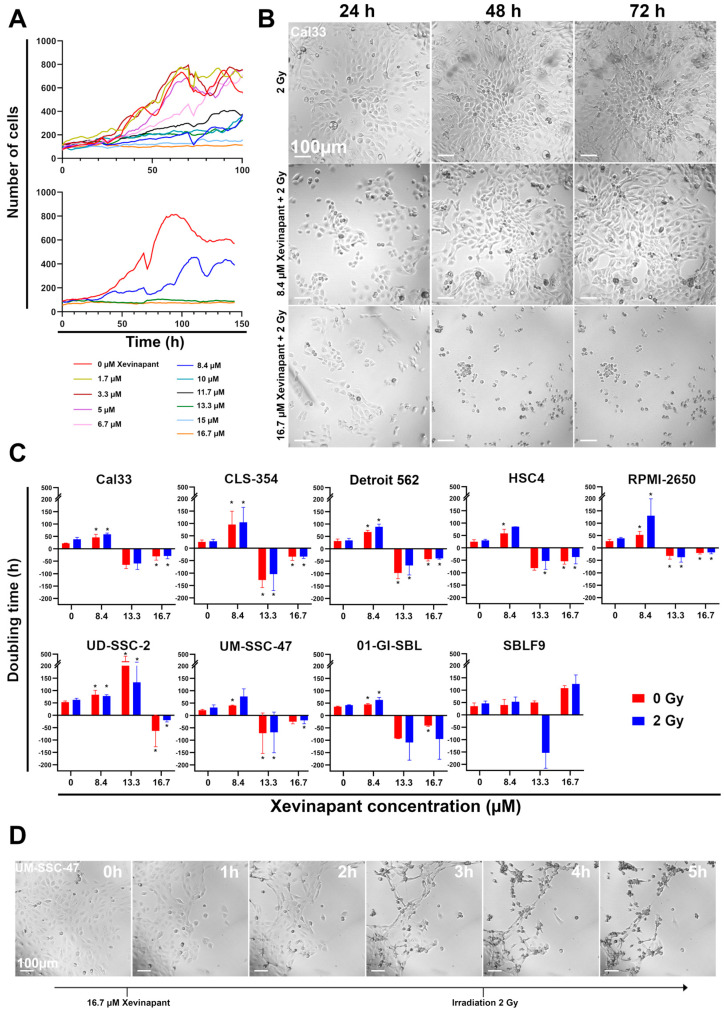
Analysis of cell growth by live microscopy: (**A**) Growth curves of HNSCC Cal33 cells with different concentrations of SMAC mimetic Xevinapant. Xevinapant was applied at 24 h and removed at 72 h. (**B**) Representative images of Cal33 cells treated with IR (2 Gy) alone and in combination with Xevinapant (8.4 µM; 16.7 µM) 24, 48 and 72 h after beginning of monitoring. Addition of Xevinapant was at 24 h. (**C**) Doubling times of all cell lines under treatment with different concentrations (0; 8.4; 13.3; 16.7 µM) of Xevinapant alone (red) and in combination with 2 Gy IR (blue). Negative doubling time is the time for the number of cells to fall to half its current level. Each value represents mean ± SD (n ≥ 3). Significance of doubling time between the Xevinapant-treated groups and the untreated, nonirradiated group, and between the Xevinapant-treated groups with IR and the group with IR alone, was determined by Mann–Whitney U test * *p* ≤ 0.05. * means that the values were compared with those of the same group (0 Gy or 2 Gy) at 0 µM Xevinapant. (**D**) Representative images of HNSCC cell line UM-SSC-47 from 1 to 5 h after application of 16.7 µM Xevinapant.

To determine an effective concentration of Xevinapant, cells were first treated with increasing concentrations from 1.7 µM to 16.7 µM alone and in combination with 2 Gy IR, and their growth was observed with a 24-well camera (Figure 2). Growth curves were then measured from these data. At lower concentrations of up to 5 µM, their growth was barely affected compared with an untreated control, while at concentrations above 8.4 µM, Xevinapant had an inhibitory effect on the growth rate (Figure 2A).

Based on these observations, three different concentrations (8.4, 13.3 and 16.7 µM) of Xevinapant were chosen to study their effect on the cells in more detail (Figure 2A,B). The doubling times of all cell lines were determined after treatment with the three different concentrations of Xevinapant alone and in combination with IR (Figure 2C). Both as a monotherapy and with irradiation, 8.4 µM Xevinapant was clearly cytostatic, with an increase in doubling time compared with the untreated or only irradiated control in nearly all cell lines (*p* ≤ 0.05). The longer doubling time shows that Xevinapant inhibits cell growth because it takes longer for the cell population to double. In the HNSCC cell lines, the increase in doubling time between the control and the fraction treated with Xevinapant ranged from 20 to 74%, both without and with IR. CLS-354 cells, which increased doubling time by 73% and 74% when treated with Xevinapant alone or combined with IR, had the most significant effect (*p* ≤ 0.05). In RPMI-2650 cells, the combination of Xevinapant and IR especially led to a strong increase in doubling time by 70% compared with IR alone, while the increase in the unirradiated fraction was only 48% after Xevinapant treatment (*p* = 0.05). The combined treatment also appeared to have a synergistic effect on Detroit 562 (*p* ≤ 0.05), HSC4, UM-SSC-47 and 01-GI-SBL cells. In these cell lines, there was a greater increase in doubling time after the combination therapy with Xevinapant and IR than after the separate administration of these two therapies. The effect of Xevinapant was the lowest, with no significant increase in doubling time in SBLF9 fibroblasts as a healthy tissue cell line. At 16.7 µM Xevinapant, the doubling time was negative in all cell lines except SBLF9, meaning that the cells did not continue to grow, but instead, the number of cells was reduced after Xevinapant treatment due to the strong cytotoxicity at this dose (*p* ≤ 0.05). This effect occurred almost immediately after administration of Xevinapant and persisted throughout the entire observation period (Figure 2D). An amount of 13.3 µM Xevinapant also resulted in a negative doubling time in most of the cell lines, except for UD-SSC-2, where growth was only strongly inhibited (*p* ≤ 0.05). However, at this concentration, the cytotoxic effect was mostly not as strong or rapid as at the highest concentration.

### 3.2. Cell Inactivation by Xevinapant in the Colony Formation Assay

**Figure 3:**
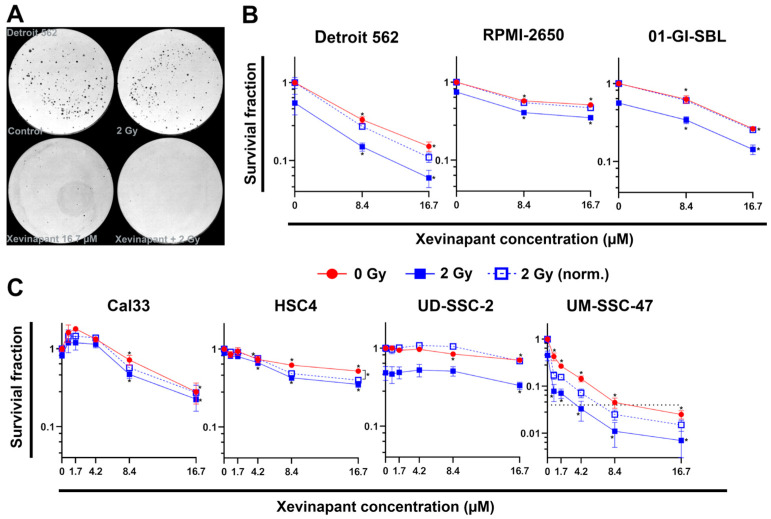
Colony formation assay of six HNSCC cell lines and one healthy oral mucosa cell line: (**A**) Representative images of HNSCC Detroit 562 cells seeded in petri dishes. Untreated cells (control), cells treated with only IR (2 Gy), with only Xevinapant (16.7 µM) and with the combination of both are shown. (**B**) Semilogarithmic plots of mean survival fraction ± SD (n = 3) after treatment with 8.4 or 16.7 µM Xevinapant. Red line shows the unirradiated and blue line the irradiated fraction at 2 Gy IR dose. Dashed line represents mean survival fraction at 2 Gy, normalized to the unirradiated group. (**C**) Semilogarithmic plots of mean survival fraction ± SD (n = 3) after treatment with different concentrations (0; 0.8; 1.7; 4.2; 8.4; 16,7 µM) of Xevinapant alone (red) and in combination with 2 Gy IR (blue). Significance of the SF between Xevinapant-treated cells and untreated control and between Xevinapant-treated cells with IR and IR alone was determined by Mann–Whitney U test * *p* ≤ 0.05. * without parenthesis means that the values are compared with those of the same group (0 Gy or 2 Gy) at 0 µM.

Noting the different effect of 8.4 µM and 16.7 µM Xevinapant, these concentrations were then used to further investigate the clonogenic ability of the cells after treatment by performing colony formation assays (Figure 3). In all cell lines, both concentrations as a monotherapy resulted in a clear decrease in the survival fraction (*p* = 0.05). In combination with 2 Gy IR, 8.4 and 16.7 µM Xevinapant clearly reduced survival compared with the treatment with IR alone in most of the cells as well (*p* = 0.05). UD-SSC-2 cells were more resistant, and the survival fraction of the irradiated cells decreased only at 16.7 µM (Figure 3A–C).

In order not to overlook any low-dose effects, four cell lines, UD-SSC-2 and UM-SSC-47, being HPV-positive cell lines, and Cal33 and HSC4, being HPV-negative cell lines, were additionally treated with lower concentrations of Xevinapant (Figure 3C). In HSC4 cells, there was a clear effect already at 4.2 µM, and in UM-SSC-47 cells already at 0.8 µM, namely reducing the survival fraction (*p* = 0.05).

The 2 Gy data were normalized to the cytotoxic effect induced without IR to study possible synergistic effects between Xevinapant and IR. In Cal33, Detroit 562, HSC4, RPMI-2650 and UM-SSC-47 cells, the combination of Xevinapant and IR tended to result in a greater reduction in survival than the two therapies given separately, but only in HSC4 cells a significance for the synergistic effect of the combined treatment could be determined. In UD-SSC-2 cells and in the healthy 01-GI-SBL cells, there was no indication for a possible supra-additive effect of the combination therapy.

### 3.3. Induction of Apoptotic and Necrotic Cell Death by Xevinapant

**Figure 4:**
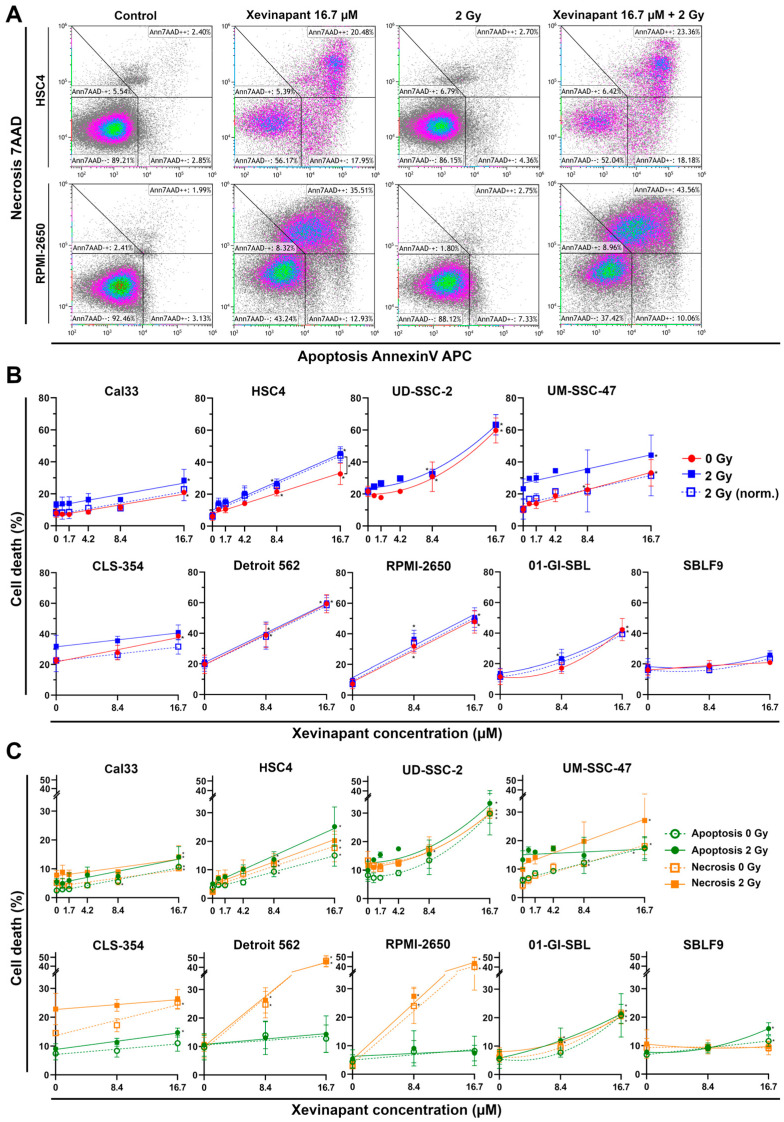
Flow cytometric cell death analysis after monotherapy with Xevinapant or in combination with 2 Gy IR: (**A**) Exemplary gating strategy of AnnexinV/7AAD staining. Ann7AAD − − cells defined as “alive”, Ann7AAD + + as “necrotic” and Ann7AAD + − as “apoptotic”. Plots of HSC4 and RPMI-2650 cells untreated (control), treated with either Xevinapant (16.7 µM) or IR (2 Gy) alone and in combination. (**B**) All cells were treated with 8.4 µM and 16.7 µM Xevinapant alone (red) and in combination with 2 Gy IR (blue). A total of 4 cell lines were additionally treated with lower concentrations of Xevinapant (0.8; 1.7; 4.2 µM). Graphs were fitted using linear regression analysis. Dashed line represents cell death at 2 Gy, normalized to the unirradiated group. (**C**) Cell death separated into apoptosis (green) and necrosis (orange). Xevinapant doses are equal to (**B**). Dashed line represents cells treated with Xevinapant alone, and straight line represents cells treated with Xevinapant and IR (2 Gy) in combination. Each value represents mean ± SD (8.4, 16.7 µM, *n* = 3; 0.8, 1.7, 4.2 µM, *n* = 2). Significance of cell death between Xevinapant-treated cells and the untreated control and between Xevinapant-treated cells with IR and IR alone was determined by Mann–Whitney U test * *p* ≤ 0.05. * without parenthesis means that the values were compared with those of the same group (0 Gy or 2 Gy) at 0 µM.

Using the identical doses of Xevinapant, cell death was measured by flow cytometry after staining the cells with AnnexinV APC and 7AAD (Figure 4). Unstained cells were defined as “alive”, positively stained cells for both agents as “necrotic” and only AnnexinV APC as “apoptotic” (Figure 4A). In six out of seven malignant cell lines, apoptotic and necrotic cell death increased linearly with increasing concentrations of Xevinapant, indicating that lower concentrations were already effective (Figure 4B). In UD-SSC-2 cells and in the healthy cell lines 01-GI-SBL and SBLF9, cell death followed a linear quadratic increase with less effect at lower concentrations. Depending on the cell line, the increase in cell death at 16.7 µM Xevinapant compared with the control at 0 µM Xevinapant ranged from 9 to 42% and was similar for the unirradiated and the irradiated fraction (*p* = 0.05). SBLF9 cells were barely affected by the treatment. The normalized data showed a clear synergistic effect between Xevinapant and IR only in HSC4 cells, where cell death increased particularly after the combination treatment (*p* = 0.05). 

Due to the ability of Xevinapant to specifically enhance apoptosis, cell death was also studied separately for necrosis and apoptosis (Figure 4C). Here, the effect of Xevinapant depended on the cell line. RPMI-2650 and Detroit 562 cells became predominantly necrotic, whereas the other cell lines showed a more equal distribution of apoptotic and necrotic cells. In Cal33, UD-SSC-2 and SBLF9 cells, apoptosis was increased slightly more than necrosis. Again, in HSC4 cells, the combined treatment of Xevinapant and IR enhanced apoptotic death in a synergistic manner, with a greater increase in cell death compared with Xevinapant and IR administered separately (*p* = 0.05). In other cell lines, there were some smaller similar trends as well, although they were not statistically significant.

### 3.4. Remaining DNA Double-Strand Breaks Studied by γH2AX

**Figure 5:**
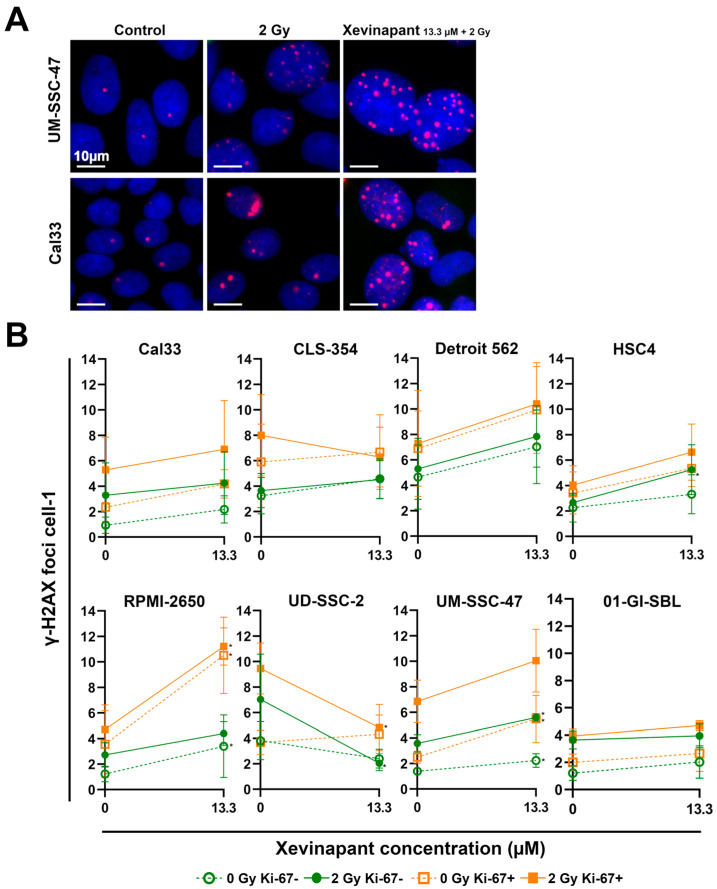
Analysis of remaining DNA DSBs after 48 h using γH2AX immunostaining assay: (**A**) Representative images of γH2AX foci in HNSCC cells Cal33 and UM-SSC-47 in untreated cells (control) 48 h after treatment with IR (2 Gy) alone or after combined treatment with IR and Xevinapant (13.3 µM). γH2AX foci are red and localized in the blue nucleus. (**B**) Number of foci per cell in each cell line after treatment with Xevinapant (13.3 µM) alone (dashed line) and in combination with IR dose of 2 Gy (straight line). Green line represents foci in Ki-67 negative cells, and orange line represents foci in Ki-67 positive cells. The average of the foci of all cells (n > 400) counted was determined in each experiment. Each value in the graph represents the total mean ± SD of at least 3 independent experiments. Significance of DNA DSBs between Xevinapant-treated cells and the untreated control and between Xevinapant-treated cells with IR and IR alone was determined by Mann–Whitney U test * *p* ≤ 0.05. * means that the values were compared with those of the same group (0 Gy or 2 Gy) at 0 µM.

An immunostaining assay for γH2AX was performed to study the effect of Xevinapant and IR on DNA damage and possible interactions with DNA damage repair in cells (Figure 5). γH2AX after 48 h represents the nonrepaired, remaining DNA double-strand breaks (DSBs), which were induced by IR or other cytotoxic agents and can therefore be used as a biomarker for DNA damage response. γH2AX is also generated during apoptotic DNA fragmentation [63,64,65]. 

While IR alone slightly induced γH2AX foci, the additional administration of Xevinapant appeared to increase the number of foci even further in nearly all cell lines. Both as a monotherapy without irradiation and in combination with 2 Gy IR, Xevinapant treatment led to DNA DSBs that could not be repaired by the cells even after 48 h (Figure 5A,B). The combined treatment appeared to have a slight supra-additive effect in HSC4 and UM-SSC-47 cells compared with the treatment with Xevinapant and IR separately. In the healthy 01-GI-SBL cells, the DNA damage repair seemed to be less affected by Xevinapant. Interestingly, in UD-SSC-2 cells, treatment with Xevinapant, especially in combination with IR, led to a decrease in γH2AX foci. As γH2AX foci can also represent stalled replication forks due to replication stress, the Ki-67 status of the cells was determined, and by measuring γH2AX only in Ki-67 negative cells, replicating cells were excluded, and γH2AX foci were more likely DNA DSBs, induced by IR and/or Xevinapant [66]. 

### 3.5. cIAP1 and XIAP Levels in Different Cell Lines

**Figure 6:**
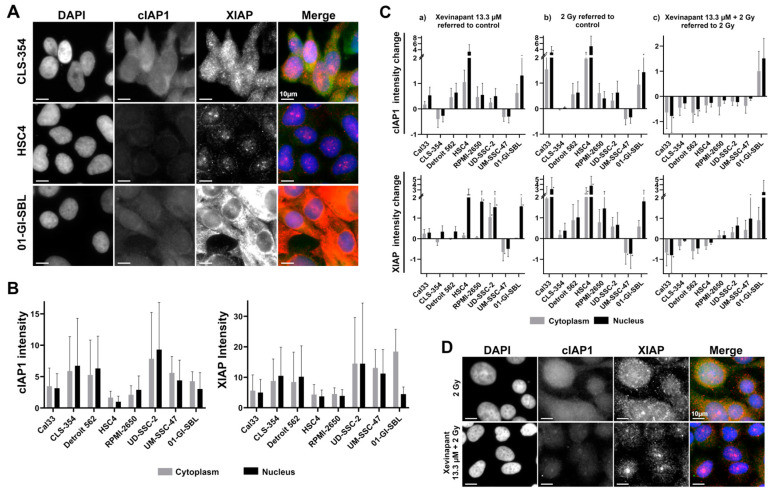
Analysis of Xevinapant target proteins cIAP1 and XIAP using immunostaining: (**A**) Representative images of untreated cells from three different cell lines (CLS-354, HSC4, 01-GI-SBL) stained with DAPI (blue), cIAP1 (green) and XIAP (red)—fluorescence microscopy image. (**B**) Background expression levels of cIAP1 and XIAP in untreated cells of seven HNSCC cell lines and one healthy cell line. Gray bar represents protein intensity in the cytoplasm, and black bar represents protein intensity in the nucleus of the cells. (**C**) Change in cIAP1 and XIAP expression levels in (**a**) cells treated with Xevinapant (13.3 µM) compared with untreated cells (negative control), (**b**) cells treated with IR (2 Gy) compared with untreated cells or (**c**) cells treated with a combination of Xevinapant (13.3 µM) and IR (2 Gy) compared with treatment with IR alone. In each experiment, the average protein intensity of all counted cells (n > 400) was determined. Each value in the graph represents the total mean ± SD of at least 3 independent experiments. Significance was determined by Mann–Whitney U test * *p* ≤ 0.05. (**D**) Representative images of Cal33 cells stained with DAPI (blue), cIAP1 (green) and XIAP (red)—fluorescence microscopy image. Top: cells after treatment with IR (2 Gy); bottom: cells treated with combination of IR (2 Gy) and Xevinapant (13.3 µM).

Xevinapant targets cIAP1 and XIAP. Both are inhibitory regulatory proteins in the apoptotic and necroptotic pathway. Therefore, the levels of cIAP1 and XIAP in various cell lines and the effect of Xevinapant alone or in combination with IR on these proteins were analyzed by immunofluorescence (Figure 6).

Depending on the cell line, different levels of cIAP1 and XIAP were found in the untreated cells. However, the distribution of the protein levels across cell lines was very similar for both cIAP1 and XIAP (Figure 6A,B and Figure S1). After treatment with IR or Xevinapant alone, cIAP1 and XIAP levels appeared to increase in most of the cell lines compared with the untreated control, except for the CLS-354 and UM-SSC-47 cells. However, compared with IR treatment alone, the combination therapy of Xevinapant and IR tended to reduce cIAP1 levels in all malignant cell lines. cIAP1 was upregulated after the combined treatment only in the healthy 01-GI-SBL cells. The degradation of XIAP after Xevinapant and IR was not as consistent, as XIAP remained upregulated in a larger number of cell lines (Figure 6C,D).

### 3.6. Association between the Levels of cIAP1 and XIAP and Cell Death

**Figure 7:**
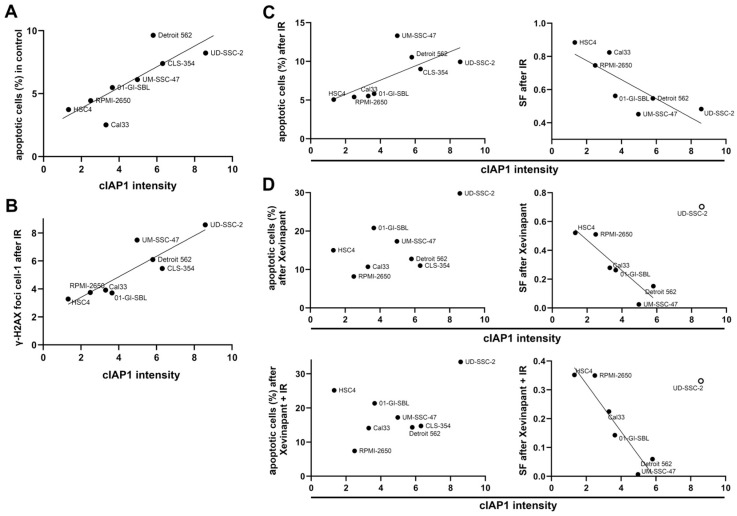
Association between cIAP1 levels and response to IR and Xevinapant therapy: (**A**) cIAP1 background levels in relation to apoptotic cells in seven HNSCC cell lines and one healthy cell line measured by AnnexinV flow cytometry. Line represents linear regression. (**B**) cIAP1 background levels associated with number of γH2AX foci per cell after 2 Gy IR. (**C**) cIAP1 background levels associated with apoptotic cells in flow cytometry and survival fraction (SF) in colony formation assay after IR. (**D**) cIAP1 background levels associated with apoptotic cells and SF after treatment with Xevinapant alone or combined with IR. The line represents linear regression. An open circle means that the cell line was excluded from the regression.

To determine whether cIAP1 or XIAP levels are a marker of sensitivity or resistance to treatment with Xevinapant, a possible correlation between the levels of these two proteins and the effect of the therapy was investigated using linear regression analysis (Figure 7 and Figure S1). Unexpectedly, cells with higher background levels of cIAP1 also had a higher rate of apoptosis measured by AnnexinV flow cytometry (Figure 7A). Remaining DNA DSBs after 48 h were analyzed to determine the cause of the increased apoptosis. An association was also found to exist between the background levels of cIAP1 and the amount of the remaining DNA DSBs in the cells after exposure to IR (Figure 7B). In addition, cells with higher cIAP1 levels also had an increase in apoptosis and a decrease in survival rates in colony formation assays after IR treatment (Figure 7C). Interestingly, there was no longer an association between cIAP1 levels and apoptosis after treatment with Xevinapant as a monotherapy or in combination with IR. However, there was still an association between cIAP1 levels and survival after treatment with Xevinapant. Xevinapant alone even appeared to be more cytotoxic than IR alone. This was especially true in cells with more cIAP1 (Figure 7D). Regarding XIAP expression, the associations between XIAP background levels and apoptosis, remaining DNA DSBs and survival in the control group and after IR were similar to those for cIAP1 (Figure S2A–C). However, in untreated cells, XIAP levels seemed to be less associated with apoptosis than cIAP1. Interestingly, there still was a slight association between XIAP levels and apoptosis after treatment with Xevinapant. The association between XIAP and survival after Xevinapant treatment was consistent with the results for cIAP1 (Figure S2D).

### 3.7. Association between the Upregulation of cIAP1 and XIAP and the Clonogenic Survival Rate

**Figure 8:**
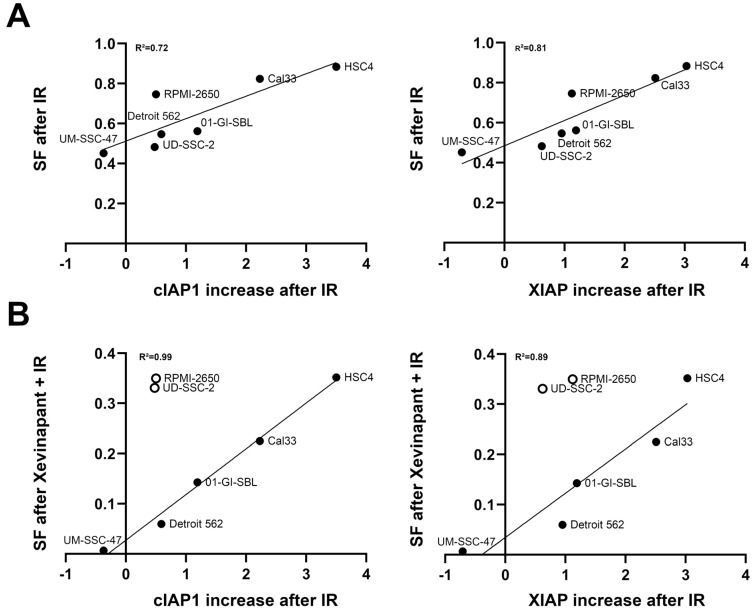
Association between upregulation of cIAP1 and XIAP levels and clonogenic survival: (**A**) Increase in cIAP1 and XIAP levels after IR plotted against the survival fraction (SF) after IR and (**B**) after the combination of IR and Xevinapant, measured by colony formation assay. Line represents linear regression analysis. An open circle means that the cell line was excluded from the regression.

cIAP1 and XIAP levels appeared to be upregulated after exposure to IR in almost all cell lines (Figure 6C). The increase in cIAP1 and XIAP levels after IR was plotted against survival rates in colony formation assays after IR and after IR in combination with Xevinapant to investigate whether the extent of this upregulation had an impact on the sensitivity of the cells to subsequent Xevinapant administration (Figure 8). Cells that upregulated their cIAP1 or XIAP expression after IR particularly strongly compared with cIAP1 or XIAP levels in the untreated control appeared to be less sensitive to irradiation and to additional Xevinapant treatment. These cells were more likely to survive than cells that did not upregulate their cIAP1 and XIAP as much (Figure 8A,B).

## 4. Discussion

The aim of this study was to investigate different effects of SMAC mimetic Xevinapant alone and in combination with IR on HNSCC cells. In all 7 HNSCC cell lines used, treatment with Xevinapant at 8.4 µM had a clear cytostatic or cytotoxic effect regarding doubling time, cell survival, apoptosis/necrosis and DNA damage repair. This effect was even more pronounced at 13.3 and 16.7 µM. In HSC4 and UM-SSC-47 cells, Xevinapant caused reduced survival, even at lower doses. Regarding the increase in DNA DSBs, it should be noted that the γH2AX foci were not stained until 48 h after treatment with Xevinapant and IR. Therefore, this slight increase in foci is already relevant, as the cells should have repaired most of their DNA DSBs caused by IR after 24 to 48 h [67]. Xevinapant therefore appears to interfere with DNA damage repair, and the remaining DSBs could lead to increased cell death. That inhibition of cIAP1 and XIAP by SMAC mimetics can lead to increased residual γH2AX foci was also described by Hehlgans et al. [68]. UD-SSC-2 cells were more resistant and less effected by Xevinapant in all assays. The magnitude of the effect of treatment was dependent on the cell line and also varied for the different parameters studied. Interestingly, the apoptotic and necrotic cell death after Xevinapant treatment, as analyzed by flow cytometry, increased in a linear manner in most of the cell lines at a very low drug dose of 0.8 µM. In contrast, in the colony formation assay, there was an effect at low doses only in the UM-SSC-47 cells. In the colony formation assay, in addition to apoptosis and necrosis, other cell inactivation mechanisms, such as mitosis-coupled death or senescence, could result in the absence of colonies. The linear increase in cell death in the flow cytometric assay led to the assumption that Xevinapant specifically affects the apoptotic and necrotic pathway, which is supported by the mechanism of Xevinapant in inhibiting IAPs. Xevinapant leads to inhibition or degradation of XIAP and cIAP1/2, followed by the activation of caspases and the noncanonical NF-kB signaling pathway, which promote apoptosis and immune response [47,48,49]. cIAP1/2 also inhibits TNF-α-induced formation of the RIP1-RIP3 death complex (necrosome), and therefore, cIAP1/2 inhibition by Xevinapant also promotes programmed necrosis [30,31,32,33,47]. This may explain why Xevinapant not only induced more apoptosis but also more necrosis in the cells. Interestingly, and in contrast to Thibault et al., in our study, Xevinapant already affected the cells as a monotherapy [47]. However, in other studies, Xevinapant also displayed effects as a single agent, which is consistent with our results [54,55]. 

As RT plays an essential role in the treatment of HNSCC, a key question for this study was whether Xevinapant provides an additional benefit to ionizing radiation. While IR alone barely affected most of the malignant cell lines studied, Xevinapant provided a strong additional benefit over IR alone, reducing cell growth and cell survival, while enhancing apoptosis, necrosis and residual DNA DSBs. The combination of Xevinapant and IR tended to be supra-additive in several cell lines, especially in HSC4 cells, indicating the possible potential of Xevinapant as a radiosensitizer. This is in line with Matzinger et al., who described the radiosensitizing activity of Xevinapant in HNSCC cells, but only when applied as a prolonged therapy [50]. Concerning the HPV status of the cells, there was no clear difference in the effect of Xevinapant on HPV-positive and HPV-negative HNSCC cells. The fact that UD-SSC-2 as an HPV-positive cell line was more resistant to treatment with Xevinapant suggests that it may have a greater effect on HPV-negative cells, but the stronger cytotoxicity on the other HPV-positive cell line, UM-SSC-47, does not support this idea. Because RT also affects the surrounding healthy tissue and can cause side effects on skin, mouth and throat, two healthy cell lines were studied to examine possible effects of Xevinapant and IR on healthy tissue cells [69,70]. The skin fibroblasts SBLF9 were clearly less affected by treatment with Xevinapant alone and in combination with IR than the malignant cell lines. This would bode well for the clinical use of Xevinapant, as it would allow the drug to target cancer cells while sparing healthy tissue. However, healthy mucosal cells 01-GI-SBL were more affected by Xevinapant than SBLF9 cells, but mostly at the highest concentration of 16.7 µM. At 8.4 µM, 01-GI-SBL cells appeared to be slightly more resistant to treatment with Xevinapant than most of the malignant cells. 

A further question in this study was whether the expression of cIAP1 and XIAP, which are targets of Xevinapant, could be used as an indicator of sensitivity/resistance to treatment with Xevinapant. Previous reports have shown that SMAC mimetics, including Xevinapant, can induce a rapid degradation of cIAP1/2 and, to a much lesser extent, of XIAP in cells [34,36,37,45,47,56]. In our study, cIAP1 and XIAP levels were not reduced in most of the cell lines, except for UM-SSC-47 and CLS-354 after treatment with Xevinapant alone. On the contrary, they seemed to be rather upregulated after monotherapy with either Xevinapant or IR, and even more so with the latter. A possible explanation could be that the cIAP1 and XIAP levels in our study were measured 48 h after treatment, and the degradation of cIAP1 and XIAP is described to happen very soon after the application of Xevinapant. Liu et al. also described a rapid depletion of cIAP1 and XIAP, followed by an increase in cIAP1 and XIAP levels in some cells after 24 h [51]. Perhaps after 48 h, the cells have increased levels of cIAP1 and XIAP as a counter-regulation to previous protein degradation and as protection against the cytotoxicity of Xevinapant or IR alone. There also was a positive association between a stronger upregulation of cIAP1 and XIAP levels after IR and better survival after treatment with IR or Xevinapant in combination with IR. In our study, only the combination of Xevinapant and IR led to a reduction in cIAP1 levels in all malignant cell lines and to a reduction in XIAP levels in some cell lines. The reason for this could be a supra-additive effect and higher toxicity of the combined treatment of Xevinapant and IR. Only 01-GI-SBL cells kept both proteins upregulated, which could be another indicator that Xevinapant affects healthy tissue to a lesser extent. 

Unexpectedly, there was an association between higher levels of cIAP1 and XIAP in the different cell lines and more apoptosis in untreated cells and after IR. The same association was also found between cIAP1/XIAP levels and remaining DNA DSBs 48 h after IR. These results lead to the possible suggestion that cells with higher levels of cIAP1/XIAP may have more difficulty in processing DNA damage caused by endogenous or exogenous stress, and that therefore, cIAP1 and XIAP are upregulated in these cells to counteract high genotoxic stress levels. Since this association did not occur between cIAP1 levels and apoptosis after treatment with 16.7 µM Xevinapant alone or in combination with 2 Gy IR, we suggest that at this concentration, cIAP1 levels could be less important, because most of the cIAP1 proteins are probably inhibited by Xevinapant, and enough caspases are activated to induce apoptosis in all cell lines. The fact that there was still a slight association between XIAP levels and apoptosis after Xevinapant treatment could be due to a different affinity of Xevinapant to cIAP1 and XIAP, or to a differentially strong effect on the extrinsic or intrinsic apoptotic pathway. Interestingly, in the colony formation assay, there was still an association between higher levels of cIAP1 and XIAP and reduced survival, even after treatment with Xevinapant. This could be in line with what we stated earlier. The apoptotic pathway seems to be particularly affected by Xevinapant, so not only the cells that have more difficulty in repairing DNA damage but also all the other cells will undergo apoptosis after treatment with Xevinapant due to IAP inhibition. However, in the other types of cell death that can be detected in colony formation assays, Xevinapant mainly affects cells that are more susceptible to stress and therefore have higher levels of cIAP1 and XIAP. Another possibility is that in cells with higher levels of cIAP1 and XIAP, Xevinapant is able to unlock more IAP-inhibited pathways, leading to increased cell death. In order to better understand these associations, they would need to be further investigated in the context of SMAC expression. These last two paragraphs are speculations derived from our findings that have not been substantiated and would therefore need to be verified in further work. 

## 5. Conclusions

The cIAP1/2 and XIAP inhibitor Xevinapant inactivates all HNSCC tumor cells at higher concentrations and has much less effect on normal tissue cells. It also appears to be a potent drug for combined treatment of HNSCC because of its possible sensitizing effect in combination with IR. Intracellular levels of cIAP1 and XIAP and increased cell death are associated. There is no association with apoptotic cell death when cIAP1 and XIAP are inhibited by Xevinapant. 

## Data Availability

The data used and analyzed during the current study are available from the corresponding author on reasonable request.

## Supplementary Materials

The following supporting information can be downloaded at: https://www.mdpi.com/article/10.3390/cells12121653/s1, Figure S1: Analysis of Xevinapant target proteins cIAP1 and XIAP using immunostaining; Figure S2: Association between XIAP levels and response to IR and Xevinapant therapy.
