# Supplementary material for: The Effect of Xevinapant Combined with Ionizing Radiation on HNSCC and Normal Tissue Cells and the Impact of Xevinapant on Its Targeted Proteins cIAP1 and XIAP
Source: Cells. 2023 Jun 17;12(12):1653. doi: 10.3390/cells12121653 (PMC10297233; doi:10.3390/cells12121653)
Supplement: Supplementary file 1 [file cells-12-01653-s001.zip › cells-2405316-supplementary.pdf]

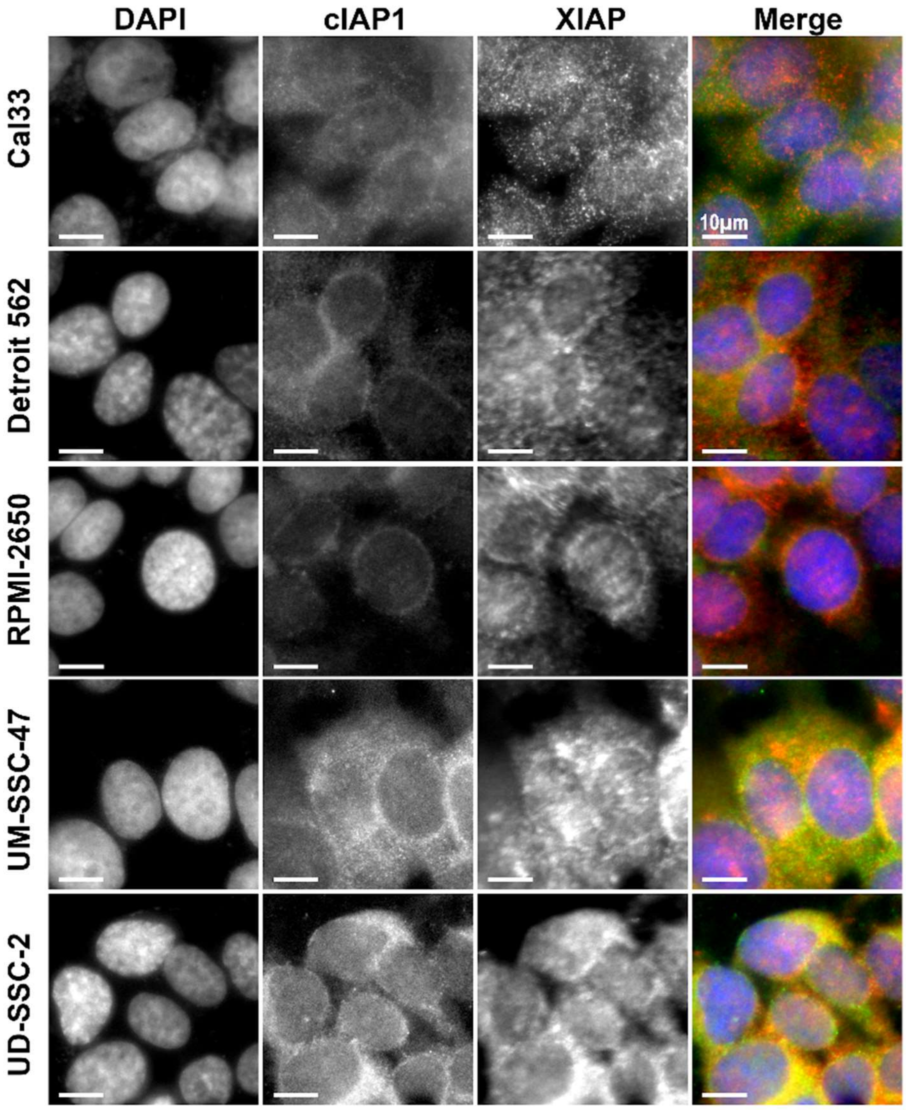

Figure S1. Analysis of Xevinapant target proteins cIAP1 and XIAP using immunostaining. Representative images of untreated cells from different cell lines (Cal33, Detroit 562, RPMI-2650, UM-SSC-47, UD-SSC-2) stained with DAPI (blue), cIAP1 (green) and XIAP (red), fluorescence microscopy image.

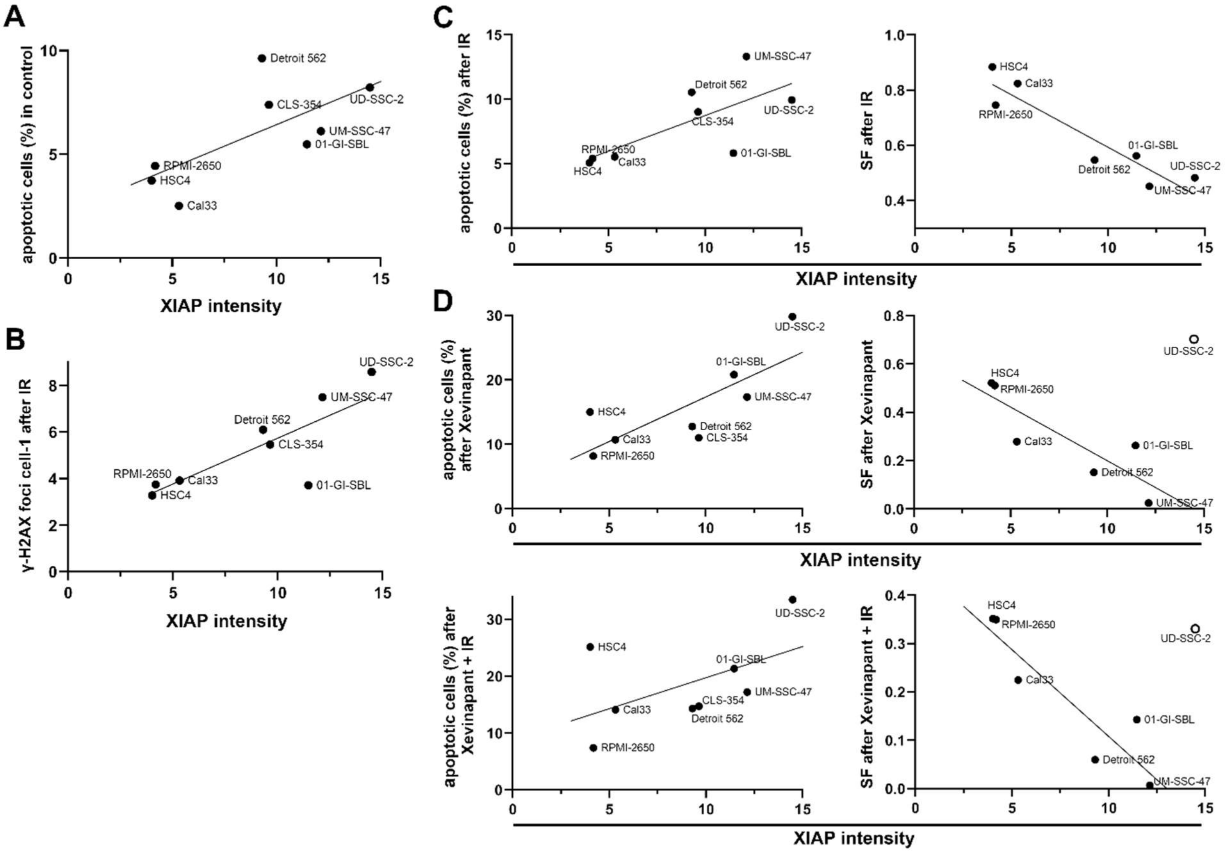

Figure S2. Association between XIAP levels and response to IR and Xevinapant therapy. (A) XIAP background levels in relation to apoptotic cells in seven HNSCC cell lines and one healthy cell line measured by AnnexinV flow cytometry. Line represents linear regression. (B) XIAP background levels associated with number of γH2AX foci per cell after 2 Gy IR. (C) XIAP background levels associated with apoptotic cells in flow cytometry and survival fraction (SF) in colony formation assays after IR. (D) XIAP background levels associated with apoptotic cells and SF after treatment with Xevinapant alone or combined with IR. The line represents linear regression. An open circle means that the cell line was excluded from the regression.
